# Allelopathy and underlying mechanism of mango (*Mangifera indica*) peel extracts on *Alexandrium catenella*

**DOI:** 10.3389/fpls.2024.1510692

**Published:** 2024-11-26

**Authors:** Yanqun Wang, Yu Zang, Wenxi Zhao, Mengxue Xu, Jie Bai, Li Li

**Affiliations:** ^1^ State Key Laboratory of Estuarine and Coastal Research, East China Normal University, Shanghai, China; ^2^ College of Environmental Science and Engineering, Ocean University of China, Qingdao, China; ^3^ Research Center of Marine Ecology, First Institute of Oceanography, MNR, Qingdao, China; ^4^ Institute of Marine Germplasm Resources, Marine Science Research Institute of Shandong Province, Qingdao, China

**Keywords:** harmful algal blooms, *Alexandrium catenella*, allelopathy, mango peel extracts, algicidal mechanism

## Abstract

Harmful algal blooms (HABs) have always been a worldwide environmental issue. The methods based on the principle of allelopathy provide a novel direction for controlling HABs; however, there are a few studies on the application of allelopathic algaecides to control harmful algae in marine environments. Here we examined the algicidal capacity of 15 fruit peel extracts with biological activity on *Alexandrium catenella*. The results displayed that the mango peel extracts (MPE) showed efficient inhibition on species growth. The algicidal rate reached 93.32 ± 0.56% at 96 h after adding 5 g/L MPE to the culture medium of *A. catenella*. Furthermore, we found that the expression of key genes involved in PSII and PSI was downregulated as well as obstructed the electron transportation in the light reaction process and the synthesis of organic matter. The blocked photosynthetic chain induced the accumulation of substantial reactive oxygen species, resulting in severe peroxidation of the membrane lipids. Simultaneously, the expression pattern of key genes involved in the fatty acid, amino acid, and peroxisome breakdown pathways was upregulated, which suggested that the synthesis and decomposition of intracellular organic matter may be in an imbalanced state. The results above indicated that oxidative damage and energy metabolism disequilibrium are two key pathways by which MPE induced algal cell death. Furthermore, several kinds of active substances and their proportion in MPE had been identified by liquid chromatography quadrupole time-of-flight mass spectrometry. It is speculated that esters may be the important component playing an algicidal effect. However, the specific substance that plays a key role in inhibiting the growth of *A. catenella* and the algicidal mechanism remain to be further studied. This study might provide a new direction in the management of HABs in the future.

## 1 Introduction

Harmful algal blooms (HABs) pose a serious threat to global marine ecosystems, which cause extensive environmental damage and economic losses and even endanger human health (Patil et al., 2024; Sakamoto et al., 2021). At present, the technology of preventing and controlling HABs mainly include physical, chemical, and biological methods. However, few methods can be applied to control HABs because of the high cost, high toxicity, and/or impracticability of most methods (Gallardo-Rodríguez et al., 2019; Jiang et al., 2020; Zong et al., 2022). Therefore, it is imperative to find an environmentally friendly method with efficient algicidal activity. The existing widely used methods (physical, chemical, and biological) are effective in preventing microalgal blooms, but most methods cannot directly modulate HABs without affecting the survival of other marine organisms (Xie et al., 2023). Therefore, the search for a very effective, environmentally feasible, and low-cost approach of managing HABs has become a key issue for the safety of the marine water environment.

The method of allelopathy with the advantages of simple operation, biodegradability, low dosage, high efficiency (Patil et al., 2024), and being environmentally friendly has attracted a number of scientists’ attention since the inhibitory effect of hydrophytes on microalgae was first reported by Hasler and Jones (1949). It has been found that the ethanol extracts of the marine brown algae *Sargassum fusiforme* inhibited the growth of *Heterosigma akashiwo*, and the antioxidant capacity and photosynthesis activity of *H. akashiwo* were significantly reduced (Sun et al., 2021). The substances with algicidal activity in the extracts are collectively referred to as allelochemicals, which may be used as a new type of algicide (Wang and Liu, 2023). In addition, some researchers have studied the allelopathic effect of fruit peel on HABs due to the presence of thousands of bioactive compounds in fruit by-products (Nath et al., 2023). Chen et al. (2019) investigated the inhibitory effect of pomegranate peel (PP) extracts on the growth of *Microcystis aeruginosa*. The results demonstrated that PP extracts can significantly inhibit the growth of *M. aeruginosa* by destroying the photosynthetic and antioxidant system of algal cells. Yan et al. (2022) investigated the possibility of converting watermelon peel into a biological resource that allelopathically inhibits *Dolichospermum flos-aquae*, and the results showed that watermelon peel aqueous extract (WMPAE) could inhibit the growth of *D. flos-aquae* by destroying the structure of algal cells, photosynthesis, and inducing oxidative stress in algal cells. Previous studies showed that allelochemicals are usually species-specific, and the allelopathic effect of the same allelopathic chemicals is highly variable between different species and even within the same species (Chen et al., 2015; Poulin et al., 2018b). This property of allelochemicals can be employed to control a specific class of HABs without harming other algal species. Therefore, it is necessary and promising to search an efficient, low-cost, and environmentally friendly method with allelopathic inhibition.

Furthermore, studies of allelopathic algicides are considered to need a substantial understanding on their mechanisms of action (Hong et al., 2008). Some researchers have elucidated the allelopathic mechanisms of various allelochemicals (Poulin et al., 2018a; Zhang et al., 2023; Zhou et al., 2017). Yu et al. (2019) found that flavonoids can influence the transcript levels of *psbD1*, *psaB*, and *rbcL* related to photosynthesis and reduce the expression of *mcyA*, *mcyD*, and *mcyH* engaged in microcystin synthesis. In recent years, researchers mainly put their attention on the study of the application and antialgal mechanism of allelochemicals on freshwater algae, and few studies have been conducted on the application of allelopathic algaecides on harmful algae in marine environments (Zhu et al., 2021). Therefore, more research is needed on the development of new and efficient allelopathic algaecides to control HABs.

*Alexandrium catenella*, a very typical and important toxin-producing dinoflagellate, leads to a huge socio-economic impact and endangers human lives during its outbreaks (Kibler et al., 2022). Considering the resource utilization of waste and economic cost, 15 prevalent fruit peel wastes that have been reported to have biological activity were employed by making them into corresponding peel extracts (Abbasi et al., 2017; Chen et al., 2019; Fujihara and Shimizu, 2003; Kato-Noguchi and Tanaka, 2003; Uehara and Baldovini, 2021; Yan et al., 2022). The optimal peel extracts capable of effectively inhibiting *A. catenella* were obtained by studying and combining the results from algicidal rate, chlorophyll fluorescence, and cell morphology of *A. catenella* exposed to different peel extracts. The change of transcription level was also studied to investigate the algicidal mechanism of the screened peel extracts. In addition, peel extracts were analyzed by liquid chromatography quadrupole time-of-flight mass spectrometry (LC-QTOF) to determine the possible compound with allelopathic activity on *A. catenella*.

## 2 Materials and methods

### 2.1 Preparation of fruit peel and peel extracts

All peels in the experiment were purchased from the market and were as follows: mangosteen (*Garcinia mangostana*), durian (*Durio zibethinus*), apple (*Malus pumila*), mango (*Mangifera indica*), passion fruit (*Passiflora edulis*), dragon fruit (*Hylocereus undulatus*), papaya (*Chaenomeles sinensis*), jujube (*Ziziphus jujuba*), grapefruit (*Citrus maxima*), orange (*Citrus reticulata*), orange (*Citrus sinensis*), lemon (*Citrus limon*), pineapple (*Ananas comosus*), banana (*Musa nana*), and pomegranate (*Punica granatum*). The method of preparation of the peel extracts was amended according to the extraction methods described by the previous researchers (Daud et al., 2010; Kato-Noguchi and Tanaka, 2003). All of the fruit peels were washed with deionized water, cut into a single layer of ca. 1 cm, and placed in a clean salver of a freeze dryer overnight. The condenser temperature was −82°C, and vacuum of 130 μB was applied, after which all of the frozen samples were sliced separately and ground into powder and stored in dark and dry conditions for further use. A part of the peel powder was taken out and mixed according to the ratio of powder (dry weight, the same as the whole text) to deionized water to 0.1 kg/L and leached for 3 days in the dark at 4°C. The rude extracts were filtered using a qualitative filter paper, stored in brown glass reagent bottles, and kept at 4°C. Each peel was disposed using the abovementioned method.

### 2.2 Microalgal bioassay

*A. catenella* (strain MEL90, GeneBan accession number MW386192) was obtained from the Marine Ecology Research Center, First Institute of Oceanography, Ministry of Natural Resources, and cultivated in modified f/2 medium (Guillard, 1975). The cultured conditions of microalgae were as follows: all samples were cultured at 20 ± 1°C under 12/12 light–dark cycle with radiance of 80 μm photon m^-2^ s^-1^ provided by a fluorescent lamp.

To screen out the peel extracts with potent inhibition effect on *A. catenella*, the algae in the exponential growth phase (5,000 cells/mL) was treated separately with the abovementioned 15 peel extracts at a final concentration of 5 g/L (the amount of raw material of peel contained in 1 L of culture medium was the same as in the whole text). A control group was set up (without the addition of peel extracts). The experiments were conducted in triplicate in 500-mL sterilized flasks containing 250 mL of medium. The effective algicidal extracts were confirmed based on the following three indicators:

The algicidal rate of different peel extracts at 48 h of the experiment was calculated. All of the samples were counted three times. The algicidal rate was calculated according to the algal cell density using the following formula:


$$Algicidal\;rate\;\left(\%\right)=\frac{N_{c}-N_{t}}{N_{c}}\times 100$$

where *N_c_* represents the algal cell density of the control group (cells/mL) and *N_t_* represents the algae cell density of the experimental group (cells/mL).

The spontaneous chlorophyll fluorescence of algal cell that can be used to characterize the growth of algal cells was detected *in situ* by the FL-3 (>670 nm) of flow cytometry (FCM, Accuri TM C6 Plus).

FCM can also be used to detect the forward-scattered light (FSC) related to cell size and the side-scattered light (SSC) indicating the fine structure and granular nature of the cell (complexity of cell contents). Each group was sampled (2 mL) and filtered by using a 300-mesh sieve silk after 48 h of experiment and then moved into the sample tube for on-machine detection (flow rate: 35 μL/s, number of cells: 5,000 cells). Gated analysis was performed based on the histograms of detected cell signals.

### 2.3 Test for effective algicidal concentration of mango peel extracts

Based on the results of the experiments above, mango peel extracts (MPE) were used for further study as the effective algicidal material. The algal cell density and the culture conditions required in the experiment groups were the same as those described in Section 2.2. MPE was added into the culture medium of *A. catenella* to make the final concentrations of the peel extracts at 1.2, 2.5, 3.8, and 5 g/L. The group without MPE was the control group, and all experimental groups and control groups were in triplicate. The algal cells of all groups were observed and counted at 48, 96, and 144 h of experiment, respectively. The algicidal rate was calculated according to the algal cell density, and the calculation method is the same as the formula that appeared above.

### 2.4 Microscopic observation of *A. catenella*

The cyto-ultrastructure of *A. catenella* was observed with a transmission electron microscope (TEM, JEOL-2100 Ltd., Japan). Next, 80 mL of treated cell culture was collected and centrifuged (4,700 r/min, 7 min, 4°C). The obtained algal cell was fixed with 3.5% glutaraldehyde at 4°C for 4 h. The cells were then rinsed three more times with 0.1 mol/L potassium phosphate buffer (pH 7.2), fixed with 1.0% OsO_4_ at 4°C for 2 h, and subsequently dehydrated using a gradient of ethanol and embedded in epoxy resin. Ultra-thin sections (70 nm) were obtained using a Reichert-Jung E ultramicrotome and double-stained with uranyl acetate and lead citrate. For the detailed operation, refer to the method described by Xue et al. (2023).

### 2.5 Physiological analysis

A total of 120 mL of treated cell culture was collected and centrifuged to obtain algal cells. The cell was stained with 2′,7′-dichlorodihydrofluorescein diacetate (DCFH-DA, 10 mM) (Solarbio, reactive oxygen species test kit) to assess the alteration of the reactive oxygen species (ROS) level of *A. catenella*. As DCFH-DA enters the algal cell and reacts with ROS, it was converted into DCF, the fluorescence intensity of which could reflect the ROS level of *A. catenella*. The treated algal cells were detected with a molecular fluorescence spectrophotometer (The excitation wavelength is 488 nm and the emission wavelength is 525 nm) (F-7100, Japan).

Malondialdehyde (MDA) representing the degree of lipid peroxidization was measured. Then, 80 mL of algal culture was harvested via gentle centrifugation (4,500 r/min, 10 min) to obtain cell pellets, and then the pellets were washed twice with sterile 0.1 M PBS (pH 7.2–7.4) (Solarbio Inc., Beijing, China). The washed algae cells were re-suspended in sterile 0.1 M PBS and sonicated at below 4°C (180 W, ultrasonic time: 1 s, rest time: 3 s) until no intact individual cell was observed with a microscope. Subsequently, the crude enzyme solution was obtained by centrifugation (12,000 rpm for 10 min at 4°C). The MDA content was measured using assay kits (Nanjing Jiancheng Bioengineering Institute, Nanjing, China) according to the manufacturer’s instructions. All assays were performed in triplicate.

### 2.6 Transcriptome analysis

According to the results of the previous experiments, 36 h was selected as the experimental node to collect samples (120 mL cell culture) for transcriptome analysis. Equal amounts of total RNA for each sample were extracted by using TRIzol (Invitrogen Life Technologies, Carlsbad, CA, USA) according to the manufacturer’s instructions. The quality and integrality of the extracted total RNA were assessed by using NanoDrop 2000 and agarose gel electrophoresis. Then, the cleaved RNA fragments were reverse-transcribed to create the final cDNA library. The cDNA library was sequenced on an Illumina platform (HiSeq X Ten, USA). FastQC was used to estimate the quality of the paired-end reads for each sample. The gene expression level was analyzed using the TPM of each gene based on gene length. Differentially expressed genes (DEGs) analysis was performed using DESeq2. Kyoto Encyclopedia of Genes and Genomes (KEGG) pathway enrichment was also completed using the cluster Profiler R software package. For pathway enrichment analysis, cluster Profiler was used to test the statistical enrichment of DEGs in KEGG pathways. All DEGs were mapped to categories in KEGG and searched for significantly enriched pathways in DEGs by comparing with the whole transcriptome background. The *p*-value was calculated using the hypergeometric test and went through multiple testing corrections. The corrected *p*-value (*q*-value) ≤0.05 was considered as the pathway that was significantly enriched.

### 2.7 Quantitative real-time PCR validation

A total of 10 DEGs were randomly selected for quantitative real-time PCR (qRT-PCR) in all groups to verify the expression patterns observed in the RNA-seq results. The reference gene, assay genes, and specific primers are listed in **Supplementary Table 1**. Reverse transcription and PCR were carried out in a system volume of 100 μL (0.5 μg RNA, 2 µL 5× TransScrip tall-in-SuperMix for qRT-PCR, 0.5 μL gDNA remover, and 90 µL nuclease-free H_2_O) and a reaction system volume of 10 μl (1 µL cDNA, 5 µL 2× PerfectStart™ Green qRT-PCR SuperMix, 0.2 µL forward primer, 0.2 µL reverse primer, and 3.6 µL nuclease-free H_2_O), respectively. Actin was chosen to be the housekeeping gene to normalize the expression levels of mRNA through the 2^−ΔΔCt^ method (Livak and Schmittgen, 2001; Wang et al., 2021).

### 2.8 Initial separation of algae-inhibiting substances in MPE

MPE was sequentially extracted with organic solvents (polarity: N-hexane < dichloromethane < ethyl acetate < water) at the ratio of 1:1 (v/v). The MPE and the organic solvent were mixed and repeatedly shaken for 20 min for extraction, and the aqueous phase and the organic phase were stratified after shaking. MPE was extracted three times per solvent, and the extracts were pooled together. The organic solvent was removed by rotary evaporation for each extraction solution, and the remaining extracts and solutions were baked in an oven at 60°C until constant weight. The powder of each phase of the same quality was weighed and rehydrated with an equal amount of water, The redissolved solution was added into the *A. catenella* medium (initial density of 5,000 cells/mL), and the final concentration was set at 5 g/L. Another group was set up to add an equal amount of MPE as the control, and the one without the addition of MPE was the blank control group. The samples were collected for observation, and the calculation of the algicidal rate was at 48 h of the experiment. The calculation method was the same as discussed above.

### 2.9 LC-QTOF analysis

Chromatographic conditions: The N-hexane phase was passed through the organic needle filter (0.22 μm pore size) to obtain the samples to be tested on the machine. Analyses were performed on a Diane Ultimate 3000 liquid chromatograph (Thermo Fisher Scientific) equipped with a Symmetry C18 (2.1 × 150 mm, 5 µm, Waters). The column temperature was 35°C. The mobile phase consisted of 0.1% (v/v) formic acid solution (eluent A) and methanol (eluent B). The flow rate was 0.2 mL/min. The gradient program was optimized as follows: T/(min): 0-2-7-15-22-25-27-29, B/(%): 10-10-35-65-85-90-10-10. The run time was 29 min. The injection volume was 5 μL.

Mass spectrometry conditions: A Bruker maXis Q-TOF (Thermo Fisher Scientific) with an electrospray ionization (ESI) system was used. Sodium formate solution was used to correct. The scan mode was full scan; scan range (m/z): 50–1,500; drying gas temperature: 180°C; drying gas flow rate: 6 L/min; atomized gas: 1.0 bar.

### 2.10 Statistical analysis

All data were presented as means ± standard deviation (SD). Visualization was performed by using Origin 2024b (Origin Lab, USA). Statistical analysis was conducted using the SPSS software package (IBM SPSS Statistics 25.0). The original data was presented as mean ± standard deviation (SD) and statistically analyzed by one-way analysis of variance, followed by Duncan’s *post-hoc* multiple comparison test using SPSS 25.0 for Windows (SPSS, Chicago, IL, USA), and *p <*0.05 was considered significant.

## 3 Results

### 3.1 MPE has optimal algicidal activity

**Figure 1:**
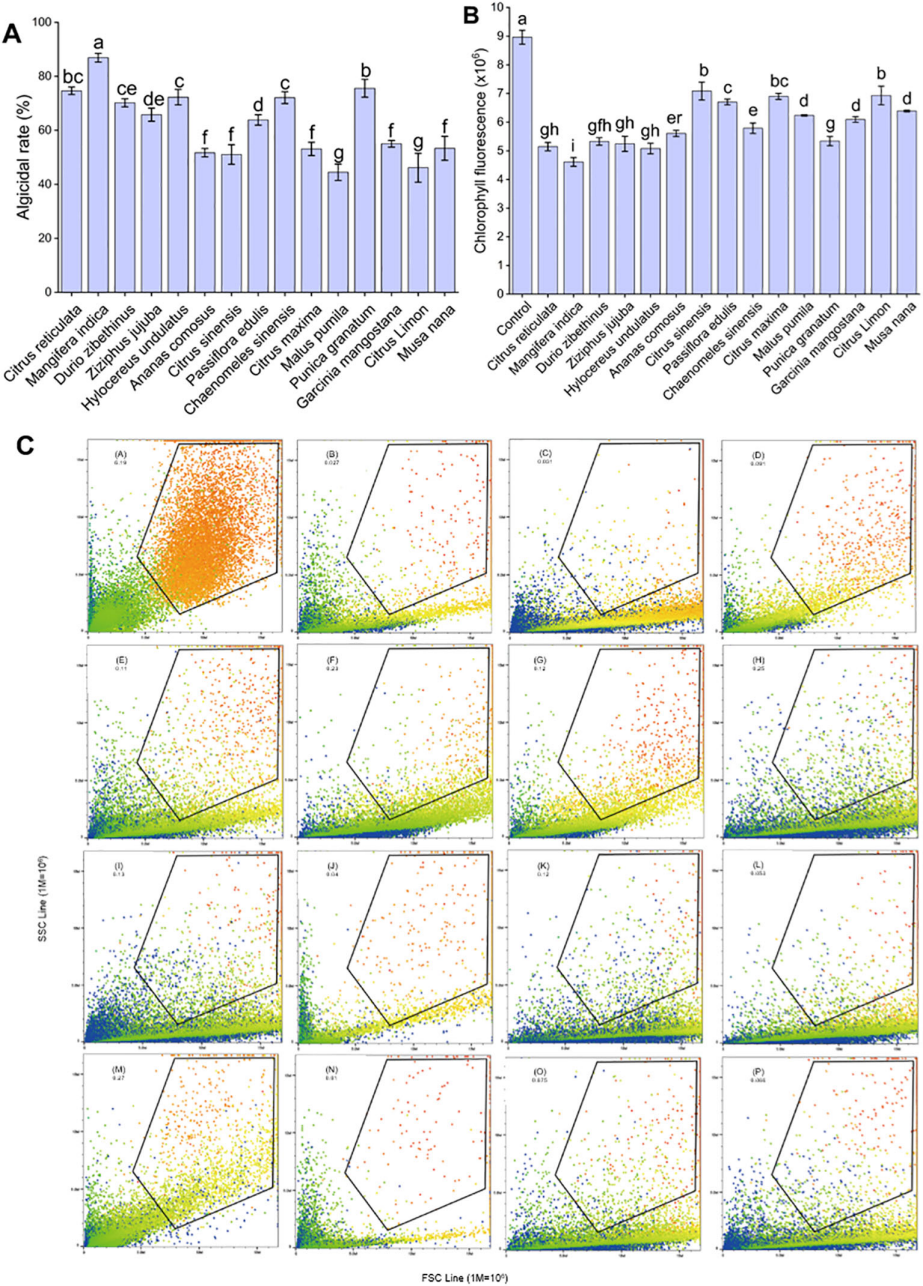
Algicidal effect of different fruit peel extracts on *A*. *catenella*. **(A)** Algicidal rate, **(B)** chlorophyll fluorescence, and **(C)** morphology of algal cells. The letters in **(C)** represent the following: control group, the peel extracts of orange (*C. sinensis*), mango (*M. indica*), durian (*D. zibethinus*), jujube (*Z. jujuba*), dragon fruit (*H. undulatus*), pineapple (*A. comosus*), orange (*C. reticulata*), passion fruit (*P. edulis*), papaya (*C. sinensis*), grapefruit (*C. maxima*), apple (*M. pumila*), pomegranate (*P. granatum*), mangosteen (*G. mangostana*), lemon (*C. limon*), and banana (*M. nana*). The data in **(C)** mean the size and granularity of the algal cells. Different lowercase letters indicate significant differences between different groups (p<0.05).

The optimal algicidal extracts were determined based on the algicidal rate, morphological characteristics, and spontaneous chlorophyll fluorescence of *A. catenella* exposed to different peel extracts (**Figure 1**). A total of 13 fruit peel extracts had an algicidal rate of more than 50% among the 15 extracts used in the experiment. Thereinto MPE exhibited the maximal algicidal effect (86.9 ± 1.61% at 48 h) (**Figure 1A**). The results of autofluorescence showed that orange peel, mango peel, durian peel, jujube peel, dragon peel, pineapple peel, papaya peel, and pomegranate peel extracts played a more significant inhibitory effect on the fluorescence value of *A. catenella* compared with the control group (*p* < 0.05) (**Figure 1B**). The mean spontaneous chlorophyll fluorescence of algal cells treated with MPE was 4.61 ± 0.15, which was the greatest influence on the spontaneous chlorophyll fluorescence of *A. catenella*. The morphological characteristics of algal cells were reflected by the values of the FSC and SSC. The results showed that the SSC values of the algal cells in the experimental groups decreased in varying degrees compared with the control group (**Figure 1C**). Given the combined results of the three indicators above, a conclusion could be obtained—that MPE had the best algicidal effect, Therefore, MPE was selected as the experimental material for subsequent research and application.

### 3.2 Optimum algicidal dose of MPE and algicidal effect

**Figure 2:**
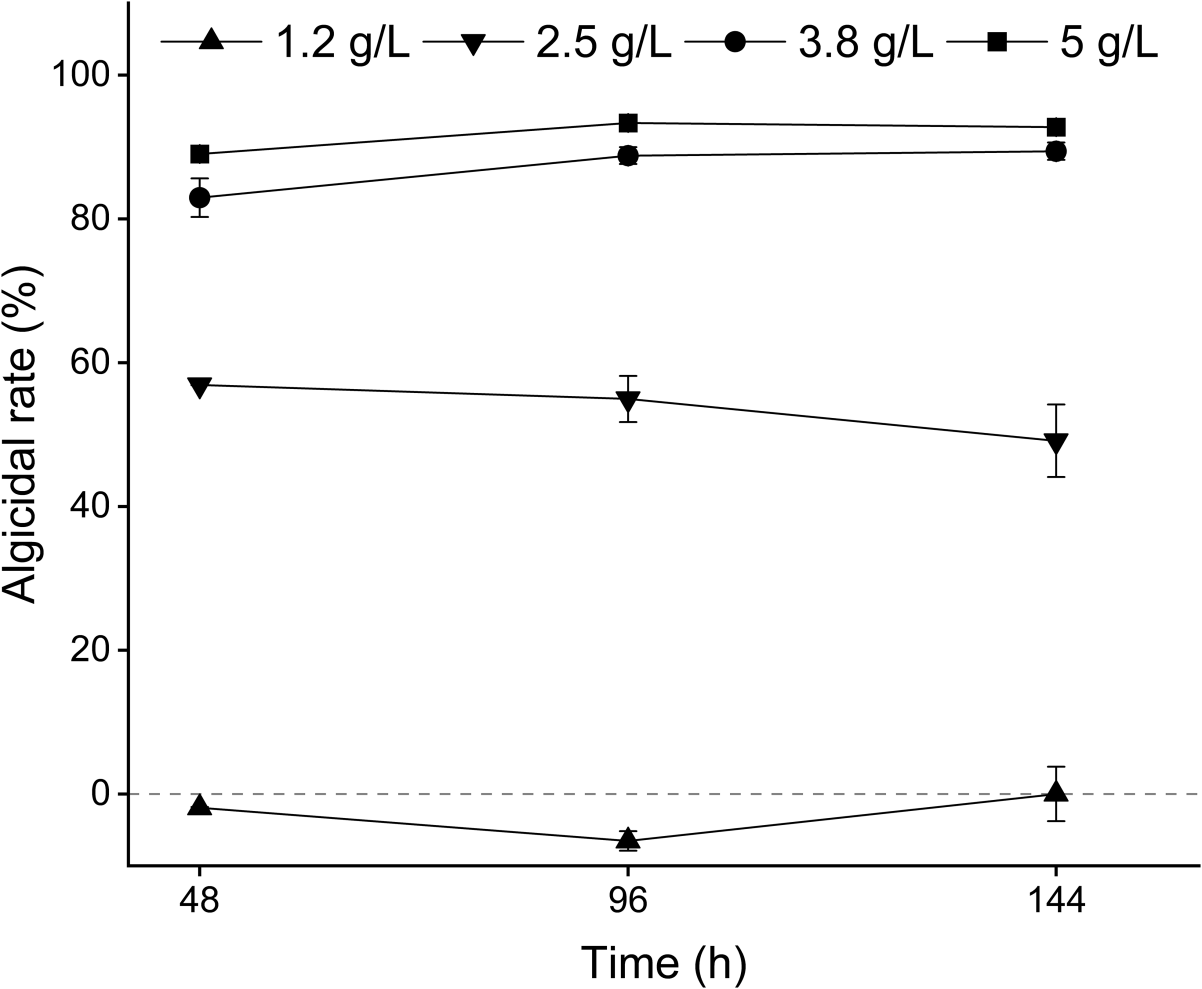
Optimum algicidal dose of mango peel extract on *A. catenella*.

Different concentrations of MPE exhibited diverse effects on *A. catenella* (**Figure 2**). At 48 h after the addition of MPE, the algicidal rates of the 1.2-, 2.5-, 3.8-, and 5-g/L groups were -1.93 ± 0.11%, 56.88 ± 0.03%, 82.95 ± 2.68%, and 89.01 ± 0.91%, respectively, and there were significant differences in the algicidal rates between the various treatment groups (*p* < 0.05). This result presented that MPE be of the property of “low promotion, high inhibition”. The algicidal rates of the 5-g/L treatment groups reached 93.32 ± 0.56% at 96 h, and a better algicidal rate remained (92.74 ± 0.89%) at 144 h (day 6), which exhibited that MPE had strong timeliness. The EC_50_ (48 h) of MPE was about 2.5 g/L.

**Figure 3:**
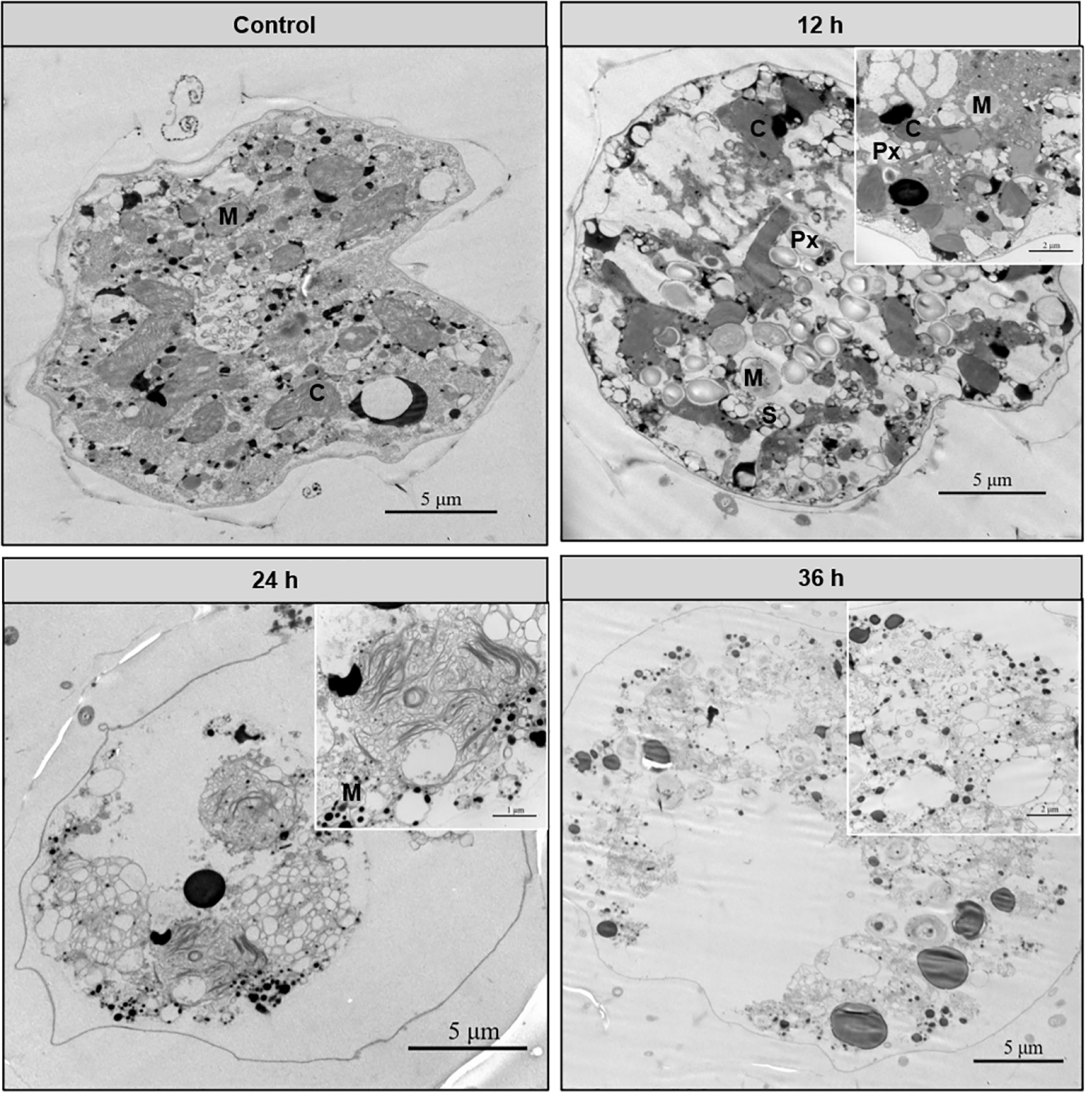
Effect of mango peel extract on the ultrastructure of algal cells. The letters in the figure represent the following, respectively: chloroplasts (C), mitochondria (M), peroxisomes (Px), and starch granules (S).

TEM was used to record the death process of algal cells at the subcellular level when *A. catenella* was exposed to MPE. Compared with the control group, the MPE-treated cells showed significant morphological differences and structural damage (**Figure 3**). *A. catenella* is an armored dinoflagellate, the normal cell of which is enclosed by a layer of thecal plates. The membrane and lamella structure of chloroplasts and mitochondria are intact and clear. Compared with the control group, the mitochondria and chloroplasts lost the lamellar structure. The boundary of the chloroplast membrane especially was blurred when the algal cell was exposed to 5 g/L MPE for 12 h. Simultaneously, a large number of starch granules and peroxisomes appeared in the cells exposed to MPE. Furthermore, the structure inside the cell underwent significant changes, and the organelles began to lyse at 24 and 36 h of the experiment.

### 3.3 Intracellular redox dynamics of *A. catenella* treated with MPE

**Figure 4:**
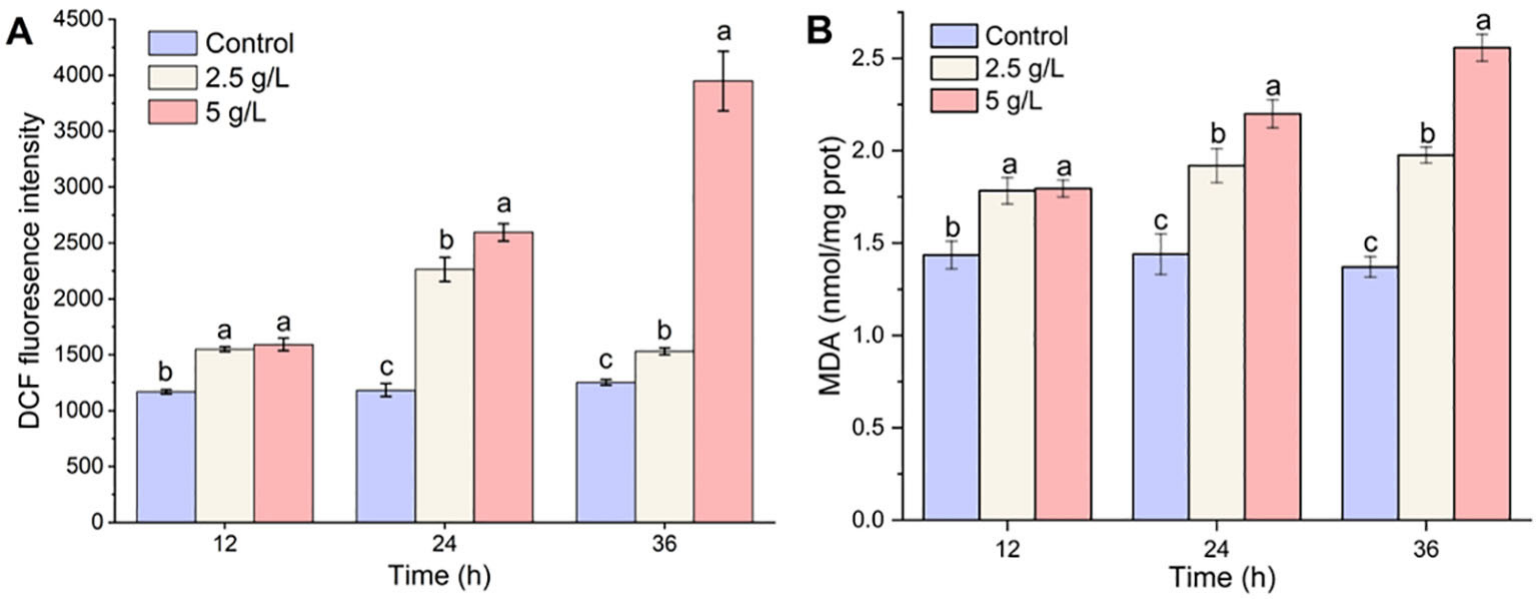
Effect of mango peel extract on reactive oxygen species **(A)** and malondialdehyde contents **(B)** in *A*. *catenella*. Different lowercase letters indicate significant differences between different groups (p<0.05).

Compared to the control, the ROS levels in the 2.5- and 5-g/L groups increased significantly at 12 h after the MPE treatment (*p* < 0.05) (**Figure 4A**). As the experiment progressed, the ROS level in the 5-g/L treatment group increased significantly at 36 h (*p* < 0.05) and was 3.15 and 2.58 times higher than that of the control group and the 2.5-g/L treatment group, respectively. The results proved that *A. catenella* produced a large amount of ROS after exposure to MPE.

Compared with the control group in which the MDA content remained at a stable status (from 1.37 ± 0.06 to 1.44 ± 0.11 nmol/mg protein), the MDA content of *A. catenella* exposure to MPE exhibited an upward trend throughout the whole experiment period (**Figure 4B**). The MDA content in the 2.5-g/L MPE treatment group increased slowly and remained stable at 36 h, while the MDA content in the 5-g/L MPE group continued to increase and reached 2.56 ± 0.07. The results demonstrated that MPE caused the peroxidation of membrane lipids in cells, which implied that there was still a vast amount of ROS accumulated in the cells.

### 3.4 Transcriptome sequencing results

**Figure 5:**
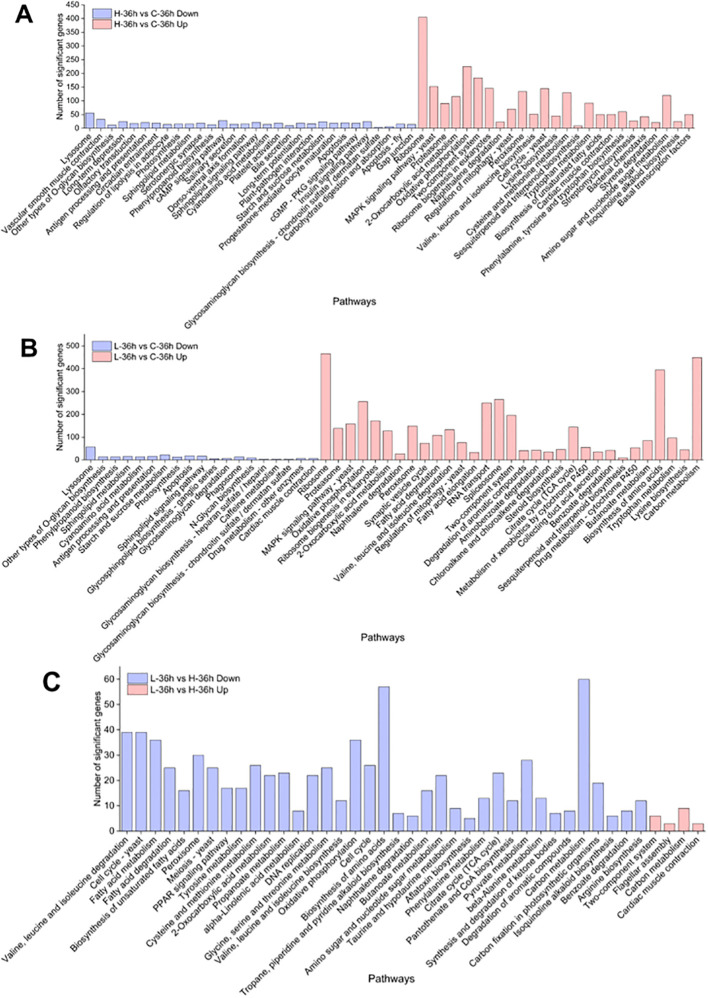
KEGG enrichment analysis of differentially expressed genes in *A*. *catenella*. **(A)** Significantly enriched pathways in 0.5 g/L vs. control (H-36 h vs. C-36 h). **(B)** Significantly enriched pathways in 0.25 g/L vs. control (L-36 h vs. C-36 h). **(C)** Significantly enriched pathways in 0.25 g/L vs. 0.25 g/L (L-36 h vs. H-36 h).

To investigate an overview of the molecular mechanisms of MPE in *A. catenella*, we constructed three comparison groups (H-36 h vs. C-36 h, L-36 h vs. C-36 h, and L-36 h vs. H-36 h). KEGG enrichment analysis was carried out to understand the biological functions and functional pathways of DEGs; the result is shown in **Figure 5**. The pathways significantly enriched in the H-36 h vs. C-36 h groups contained the following (*q*-value ≤0.05): tryptophan metabolism, proteasomes, peroxisome, cysteine and methionine metabolism, etc., were significantly upregulated (*q*-value ≤0.05). On the contrary, photosynthesis, starch and sucrose metabolism, and carbohydrate digestion and absorption pathways showed a significantly downward regulation (*q*-value ≤0.05). In addition, the peroxisome pathway was significantly upregulated (*q*-value ≤0.05). Compared with the upregulated pathway that was significantly enriched in the H-36 h vs. C-36 h group, the citrate cycle (TCA cycle), valine, leucine, and isoleucine degradation, fatty acid degradation and amino acid biosynthesis, etc., were additionally enriched in the L-36 h vs. C-36 h group. However, in the enrichment results of the L-36 h vs. H-36 h group, glycine, serine, and threonine metabolism, phenylalanine metabolism, valine, leucine, and isoleucine degradation, tyrosine metabolism, fatty acid metabolism, fatty acid degradation, peroxisome, cysteine, and methionine metabolism, etc., were significantly downregulated (*q*-value ≤0.05).

### 3.5 Analysis of DEGs associated with photosynthetic and antioxidant system and qRT-PCR validation

**Figure 6:**
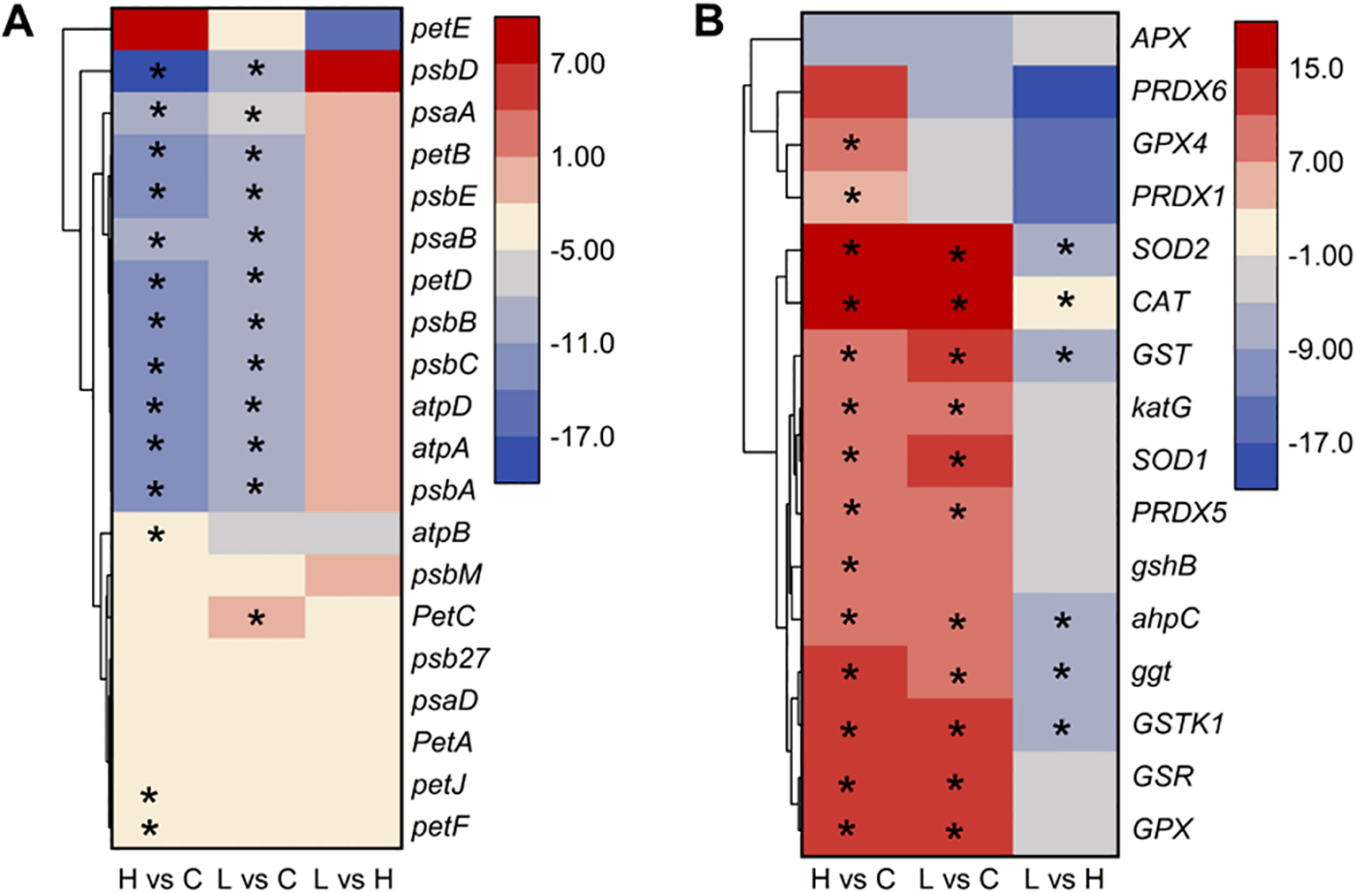
Heatmap of partial differentially expressed genes involved in photosynthetic **(A)** and antioxidant system **(B)** in *A*. *catenella* exposed with mango peel extract. The color scale in the figure represented log_2_(fold change) of genes based on hierarchical clustering and comparison of control and treatment samples. Statistical significance is indicated by * (*q*-value ≤0.05). 0.5 g/L vs. control (H vs. C), 0.25 g/L vs. control (L vs. C), 0.25 g/L vs. 0.5 g/L (L vs. H).

We furtherly analyzed the regulation and expression trends of 36 DEGs involved in the photosynthetic and antioxidant system of *A. catenella* from the transcription level. The specific results are presented in **Figure 6**. The result about the photosynthetic system showed that there were no significant DEGs found in the L-36 h vs. H-36 h group, which indicated that a low dose of MPE could significantly affect and inhibit the expression of photosynthesis-related genes of *A. catenella* (*q*-value ≤0.05). This study found that, of the 28 DEGs involved in photosynthesis, 27 DEGs were downregulated in the H-36 h vs. C-36 h group, of which 14 were significantly different (*q*-value ≤0.05). The expression levels of *psbA* gene encoding the D1 protein and *psbD* gene encoding the D2 protein were significantly downregulated (*q*-value ≤0.05), and the same tendency occurred in the result of genes (*psbB* and *psbC*) encoding core antenna complexes. Furthermore, the reaction center pigment proteins (*psaA* and *psaB*) of PSI are also significantly downregulated (*q*-value ≤0.05). The gene *petF* encoding ferredoxin and cytochrome b6 (*petB*) in the photosynthetic chain of the photosynthetic system were significantly downregulated (*q*-value ≤0.05).

In the result of analyzing DEGs involved in the antioxidant system, the expression of *GSR* gene associated with the deoxidation of GSSG to GSH was upregulated. *SOD1* (Cu–Zn) and *SOD2* (Fe-Mn) gene-encoded SOD, *CAT* gene-encoded CAT, *GPX* gene-encoded GPX, and *GSR* gene-encoded glutathione reductase were all significantly upregulated in the treatment group (H-36 h and L-36 h) (*q*-value ≤0.05). We also found *PXMP4* (peroxisome membrane protein 4) and *MPV17* (mitochondrial inner membrane protein *MPV17*) genes to be significantly upregulated in the treatment group (*q*-value ≤0.05).

The results of the qRT-PCR validation are shown in **Supplementary Figure 1**. Overall, nine DEGs had the same trend in RNA-seq and qRT-PCR, and one DEG had a different pattern in RNA-seq profiling of control and treatment samples. The 90% compliance rate between RNA-seq and qRT-PCR indicates that RNA-seq has high accuracy, indicating that the identified pathway and candidate DEGs are reliable pathways for mango peel leaching effect.

### 3.6 Analysis of other pathways that were significantly enriched in *A. catenella*

**Figure 7:**
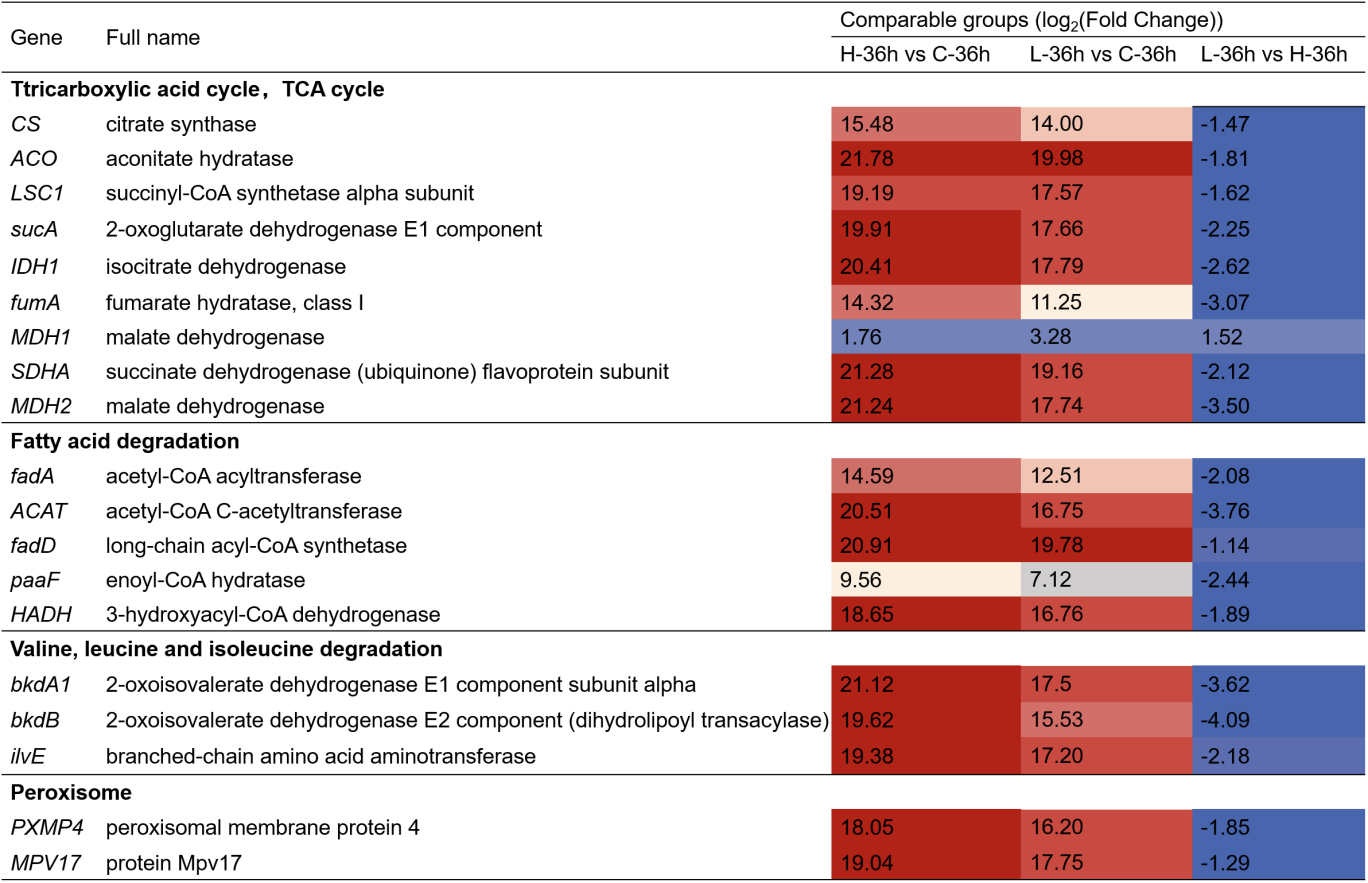
Heatmap showing the key differentially expressed genes (DEGs) involving the other pathway in *A. catenella* exposed with mango peel extract. The colors in the table represent log_2_(fold change) of DEGs. H-36 h vs. C-36 h denotes 0.5 g/L vs. control at 36 h; L-36 h vs. C-36 h denotes 0.25 g/L vs. control at 36 h; L-36 h vs. H-36 h denotes 0.25 g/L vs. 0.5 g/L at 36 h.

We also found that amino acids and fatty acids in *A. catenella* were gradually degraded with the increase of MPE according to the KEGG enrichment result. We further analyzed the pathways concerning the fatty acid and amino acid degradation. The specific information is displayed in **Figure 7**. The results showed that 108, 133, and 149 genes were separately involved in fatty acid degradation. Valine, leucine, and isoleucine degradation and peroxisome were significantly upregulated in L-36 h vs. C-36 h group (*q*-value ≤ 0.05). In the comparison of L-36 h and H-36 h, the pathways of fatty acid metabolism, fatty acid degradation, valine, leucine, and isoleucine degradation, and peroxisome were downregulated. There were 36, 25, 39, and 30 genes that were significant in the pathways mentioned above, respectively (*q*-value ≤0.05), which demonstrated that organic substances such as amino acids necessary for the growth of algal cells were more utilized in the H-36 h group.

### 3.7 Algicidal capacity of each extraction phase of MPE

**Figure 8:**
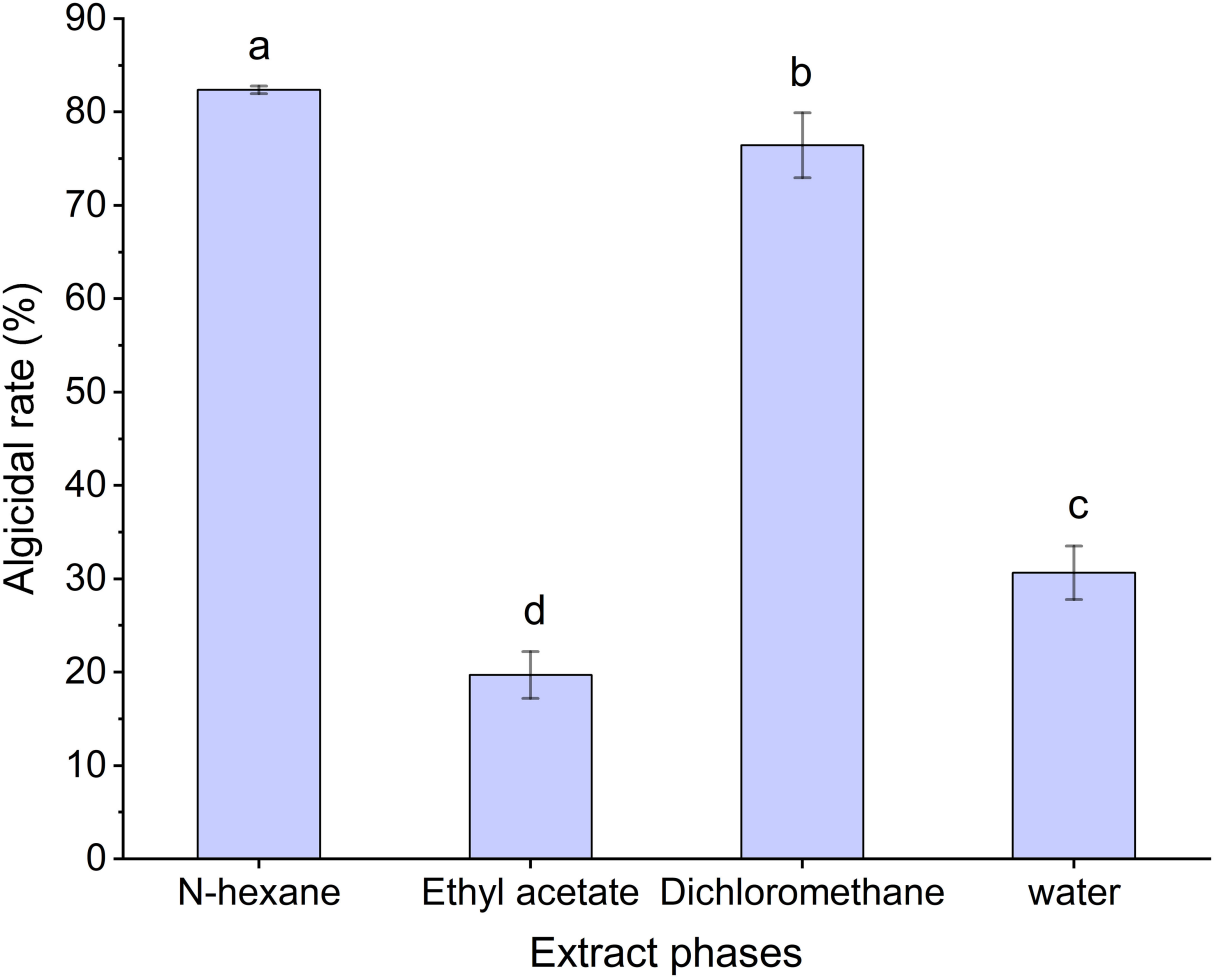
Algicidal rate of the different extraction phases of mango peel extract. Different lowercase letters indicate significant differences between different groups (p<0.05).

The algicidal rates of the different extraction phases from MPE are shown in **Figure 8**. The results showed that the N-hexane phase had the highest algicidal rate (82.38 ± 0.41%), which implied that the main algae-inhibiting substances in the MPE may be in the N-hexane phase, and the N-hexane extraction component was used for the next experiment.

### 3.8 Major algal inhibitory components in MPE based on LC-QTOF analysis

The results of the LC-QTOF analysis showed that there were 11 phytochemical constituents in the N-hexane extracts (**Supplementary Table 2**). The main phytocomponents of MPE were analyzed by their peak time (Pt), name of compounds (NCs), molecular formula (MF), mass-to-charge ratio (m/z), and peak area (PA, %). The main substances detected comprised seven esters, one aromatic acid, one phenolic compound, one amino acid, and one sugar. The main composition observed was 3-O-talopyranosylmannopyranoside, which presented the highest intensity and peak area (4.69%), followed by amino acid (4.57%). However, esters were the most abundant, and their total peak area was the highest (9%).

## 4 Discussion

In this study, the peel extracts with simple operation, low economic cost, and high efficiency in inhibiting *A. catenella* were discovered. The antialgal effect of the peel extracts was revealed from three indicators: algicidal rate, chlorophyll autofluorescence, and cell morphology. Our experimental results pointed out that MPE has shown efficient inhibition on the growth of *A. catenella*. The algicidal rate reached 93.32 ± 0.56% after adding 5 g/L MPE to the culture medium of *A. catenella* for 96 h. Moreover, MPE exhibited a low-promotion and high-inhibition mode. We speculated that a low concentration of MPE stimulated algal cells to increase their physiological activity, resulting in an effect that is opposite to that of high-dose stimulation. This is the first time that the inhibitory effect of MPE on *A. catenella* was discovered.

The TEM results showed that a large number of starch granules and peroxisomes appeared in the cells at 12 h of the experiment. Starches are the main storage form of energy substances. Peroxisomes contain massive oxidative enzymes, such as catalase, D-amino acid oxidase, and acyl-CoA oxidase, which have the function of metabolizing hydrogen peroxide and β-oxidation of fatty acids (Buléon et al., 1998; Reumann and Bartel, 2016). Therefore, the increase of peroxisomes leads to the digestion and breakdown of energy substances to supply energy for the cells. Our result demonstrated that the organelles in the cells had been completely dissolved by MPE at 36 h, leaving only part of the membrane structure of the organelles. So, how do the algicidal substances in MPE destroy algal cells step by step?

**Figure 9:**
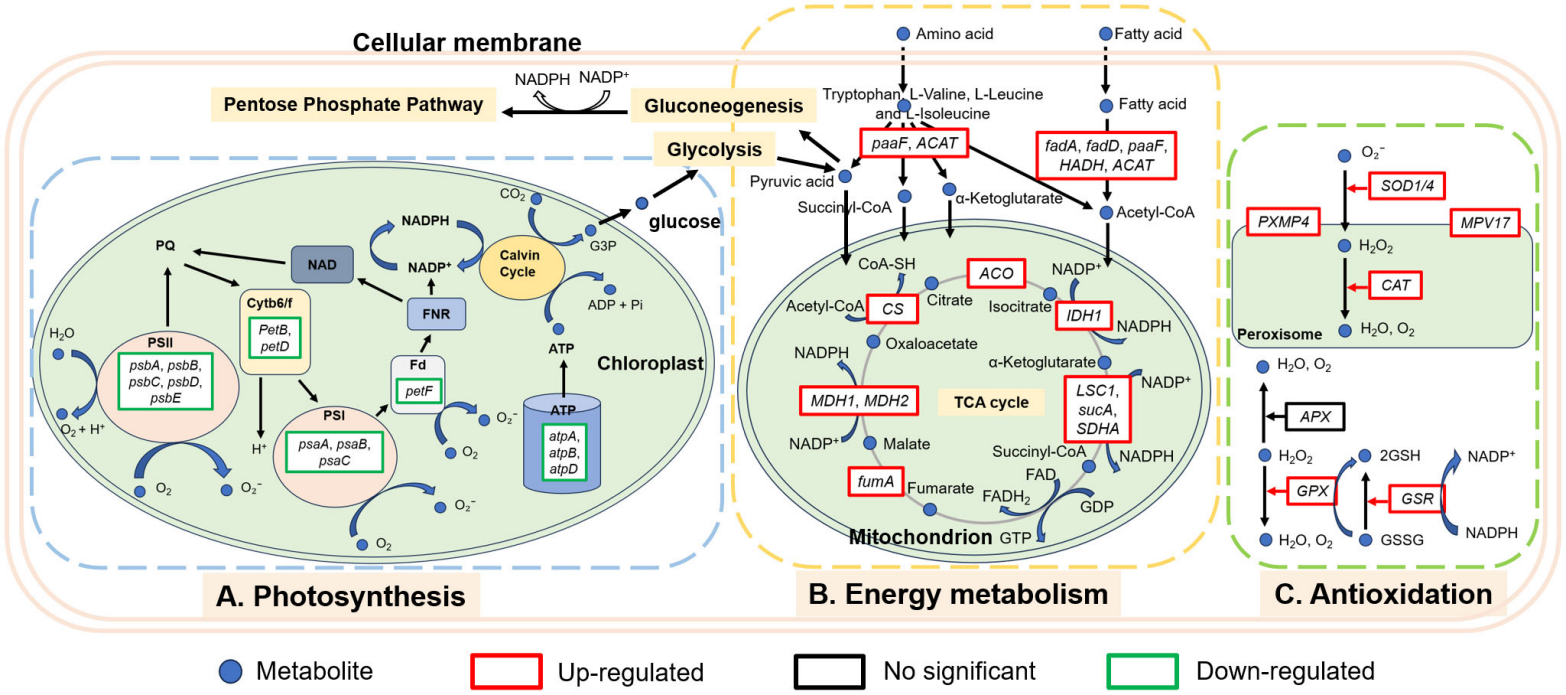
Overview of the algicidal mechanism of mango peel extract on *A. catenella*.

In order to more intuitively observe the algicidal mechanism of MPE and discover the underlying algicidal pathways, a 36-h node was selected for further analysis of the transcriptome level according to the abovementioned results. Photosynthesis is the sum of a series of complex metabolic reactions that underlie algae survival (Yu et al., 2019). KEGG enrichment analysis displayed that a large number of DEGs involved in photosynthesis were found, and there were no significant DEGs about photosynthesis in the L-36 h vs. H-36 h group, which indicated that a low dose of MPE could significantly affect and inhibit the expression of photosynthesis-related genes of *A. catenella* (*q*-value ≤0.05). This also demonstrated that the photosynthetic system is sensitive and susceptible to external stress. PSII and PSI are the reaction center complexes that drive the light reactions of photosynthesis (Eom et al., 2022). The proteins encoded by the *psbA* and *psbD* genes are key proteins of the reaction center of PSII. These proteins can bind to all redox active components involved in electron transfer and participate in the PSII repair cycle through degradation and resynthesis (Shao et al., 2009; Mulo et al., 2012). The results showed that the genes *psbA* and *psbD* were significantly downregulated (*q*-value ≤0.05), and the genes (*psbB* and *psbC*) encoding the core antennae complex were also significantly downregulated (*q*-value ≤0.05), which directly interfered the normal progress of the PSII light-harvesting complex and photoreaction center. Furthermore, the pigment proteins in the photoreaction center (*psaA* and *psaB*) of PSI were also significantly downregulated (*q*-value ≤0.05), resulting in the malfunction of PSI. Both the hampered PSI and PSII will inevitably lead to the disruption of the electron transport chain. According to the analysis results, the genes *petF* and *petB* encoding ferredoxin and cytochrome b6, respectively, in the electron transport chain of the photosynthetic system were significantly downregulated (*q*-value ≤0.05). Phosphorylation, which is coupled to the electron transport chain, was similarly inhibited. All of the results above indicated that MPE restrained the entire photosynthetic system and lead to the obstruction of organic matter synthesis of *A. catenella* (**Figure 9**).

Reductions or blockades of the electron transfer rates would lead to electron accumulation (Lu and Vonshak, 2002; Qian et al., 2012). The oxygen molecule reacting with excess electrons would lead to ROS production (Park et al., 2006; Qian et al., 2010; Schmitt et al., 2014). This was confirmed by the determination of ROS content in this study. Transcriptome analysis also showed that a large number of genes encoding enzymes involved in an antioxidant effect are significantly upregulated in the treatment group (H-36 h and L-36 h) to protect algal cells from damage by ROS (*q*-value ≤0.05). *SOD1* (Cu–Zn) and *SOD2* (Fe–Mn) gene-encoded SOD, *CAT* gene-encoded CAT, *GPX* gene-encoded GPX, and *GSR* gene-encoded glutathione reductase were all significantly upregulated in the treatment group (H-36 h and L-36 h) to protect algal cells from damage by ROS (*q*-value ≤0.05). We also found that *PXMP4* (peroxisome membrane protein 4) and *MPV17* (mitochondrial inner membrane protein *MPV17*) genes were significantly upregulated in the treatment group (*q*-value ≤0.05). *PXMP4* encoded a peroxisome membrane protein (PMP) that combines with *PEX19* (peroxisomal biogenesis factor 19) to target the peroxisome membrane (Sacksteder et al., 2000). The M-LP protein (*Mpv17*-like protein) encoded by the *MPV17* gene is located on the peroxisome membrane and participates in ROS metabolism and the regulation of genes encoding peroxisome enzymes (e.g., SOD, CAT, and GPX, et.) (Iida et al., 2006). However, the increasing MDA content in the treatment group hinted us that the large amount of ROS in the cell was still imbalanced. The excessive ROS could convert membrane lipid into toxic peroxides to destroy the biological membrane. Meanwhile, the MDA content would increase (Davey et al., 2005; Rizvi and Khan, 2018). Therefore, ROS-induced oxidative damage may be one of the pathways by which MPE inhibits the growth of *A. catenella*.

In addition, we found that the amounts of genes involved in the degradation pathway of amino acids and fatty acids in *A. catenella* were upregulated in the L-36 h vs. C-36 h group, and more genes were enriched in H-36 h. Similarly, the TCA cycle pathway was significantly enriched (*q*-value ≤0.05), in which the key genes involved were significantly upregulated (*q*-value ≤0.05), which demonstrated that organic substances necessary for the growth of algal cells were more decomposed and utilized. Simultaneously, the peroxisome pathway containing a large number of genes encoding antioxidant enzymes was significantly enriched (*q*-value ≤0.05). Peroxisome is the site of lipid decomposition and therefore can provide energy for other metabolic pathways when photosynthesis and other energy metabolism pathways are inhibited (Su et al., 2018; Zolman et al., 2000). Fatty acids can be decomposed by β-oxidative to produce acetyl-CoA, which could enter the TCA cycle for further energy release (Ghisla, 2004; Stumpf, 1969). The amino acids could also be deaminated by transamination reaction to form the corresponding acetyl-CoA, α-ketoglutaric acid, pyruvic acid, succinyl-CoA etc., which can be used in subsequent enzymatic reactions to maintain mitochondrial respiration and ATP synthesis. Moreover, the intermediates produced by the breakdown of amino acids can be converted into sugars or fats as a reserve of energy (Brand and Edsall, 1947).

In order to preliminarily determine the nature and complexity of the compounds present in MPE, MPE was extracted using three typical extractants of different polarity values (ethyl acetate, N-hexane, and dichloromethane). The results of algae inhibition of the three organic phases showed that the N-hexane phase had the highest algicidal activity (82.38 ± 0.41%); therefore, the N-hexane extract was used for further compositional analysis. LC-QTOF analysis exhibited that only one type of sugar and amino acid, respectively, was detected, but their intensity and proportion are relatively high. The peak area was 4.69% and 4.57%, respectively, and both of them were the main components in MPE. In addition, there are seven esters in the N-hexane extract, and their total peak area reached 9%. In the available studies, many esters were found to have algicidal properties, such as cholesteryl oleate, ethyl palmitate, and ethyl 2-methylacetoacetate (Zhu et al., 2021), Among the algicidal esters, ethyl 2-methylacetoacetate was found to be particularly effective in controlling algae with EC_50_ values of 0.49 and 0.65 mg/L against *Chlorella pyrenoidosa* and *Microcystis aeruginosa*, respectively (Men et al., 2007). In addition, phenolic compounds also had algae-inhibiting activity in numerous studies on algae-inhibiting active ingredients (Zuo et al., 2016). Murray et al. (2010) showed that three phenolic algicidal agents in barley straw had different algicidal activities against *C. pyrenoidosa*, *M. aeruginosa*, and *Scenedesmus*, with 2-phenylphenol having the highest algicidal activity. In this study, the phenolics detected were low, and their properties need to be verified and analyzed in further experiments. Combining the results of the compositional analysis in this study, it is presumed that the major algicidal compounds in MPE are esters, but further analysis and validation of their algicidal activity are required.

## 5 Conclusions and future research and application perspectives

In this study, MPE with potent algicidal activity were screened out. Combining the physiological–biochemical results and the transcriptional analysis, the overview of algicidal mechanism of MPE on *A. catenella* is clear. First, the photosynthetic system on which autotrophic algae rely heavily was disrupted by MPE, which induced the accumulation of a large amount of ROS and caused severe oxidative damage to the cell membrane. Second, a large number of the organic matters (amino acids and fat acids) necessary for the growth of algal cells were decomposed and utilized to support the normal operation of cellular physiological activities, and the imbalance of organic matter decomposition and synthesis led to the gradual collapse of algal cells, finally inducing the death of algal cells. Ester compounds were found to be the most abundant and proportionally highest in MPE, followed by sugar and amino acid. In summary, the MPE with efficient algicidal effect are highly promising to control HABs because it has the characteristics of low cost and minimal impact on the treated environment. This algicidal method is also a kind of resource reuse. Further studies on the current topic are needed to analyze specific algicidal substances in the MPE using reliable methods and to assess their ecological safety.

## Data Availability

The RNA-Seq raw sequence data presented in the study are deposited in the National Center for Biotechnology Information (NCBI) Sequence Read Archive (SRA) repository. The names of the repository/repositories and accession number(s) can be found below: https://www.ncbi.nlm.nih.gov/sra/PRJNA1186926.
