# Supplementary material for: Allelopathy and underlying mechanism of mango (Mangifera indica) peel extracts on Alexandrium catenella
Source: Front Plant Sci. 2024 Nov 26;15:1510692. doi: 10.3389/fpls.2024.1510692 (PMC11628311; doi:10.3389/fpls.2024.1510692)
Supplement: Supplementary file 1 [file DataSheet1.docx]

Supplementary Material

# 1 supplementary Figures and Tables

# 1.1 supplementary Figures

**
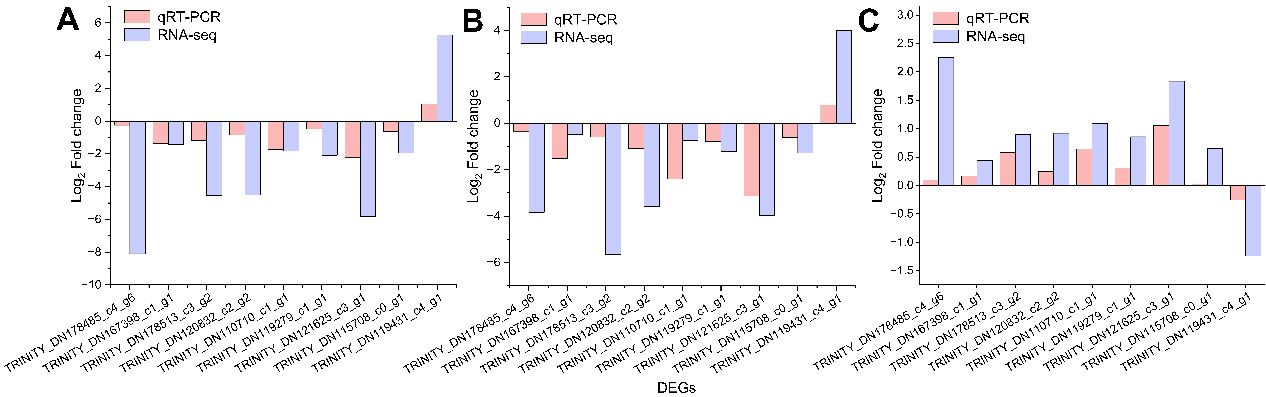
**

**Supplementary Figure 1.** Expression of partial DEGs in RNA-seq and qRT-PCR. (A) H-36 h vs C-36 h (0.5 g/L vs Control), (B) L-36 h vs C-36 h (0.25 g/L vs Control), (C) L-36 h vs H-36 h (0.25 g/L vs 0.5 g/L).

## 1.2 supplementary Tables

**Supplementary Table 1.** Genes and references used for fluorescence quantitative PCR.

| Gene | Primer | Primer sequence (5’→3’) |
| --- | --- | --- |
| ACT（reference gene） | Forward | TTCCGGCGATGTACGTTG |
|  | Reverse | TAGGCACAGTGTGTGACACGC |
| TRINITY_DN120832_c2_g2 | Forward | CAGAGAAGATAGCAATTACAGC |
|  | Reverse | CTGTGGATCTAGGATTACATGATAC |
| TRINITY_DN121625_c3_g1 | Forward | ACACGCAGAAGCCTTTA |
|  | Reverse | ATCTGAAGAGGACAAGGATATG |
| TRINITY_DN119431_c4_g1 | Forward | CCAAGATCCAGGACAAGG |
|  | Reverse | TTTGGATGTTGTAGTCCGAGA |
| TRINITY_DN178485_c4_g6 | Forward | GAACCTGTAGCAGGTAGTC |
|  | Reverse | TGAACACCTATTGCATTAGAGC |
| TRINITY_DN167398_c1_g1 | Forward | CCCTAAGCAAGGTGCAAC |
|  | Reverse | CTGATGTCGCCGTATGTG |
| TRINITY_DN178513_c3_g2 | Forward | TCAAGGGTCTCCAGAAGT |
|  | Reverse | ATCCTGTTCCACCTCCAA |
| TRINITY_DN110710_c1_g1 | Forward | GATCAGCTAATTCATTGCTGG |
|  | Reverse | CCATCAGCTTCGAGGAC |
| TRINITY_DN119279_c1_g1 | Forward | CGACATGAAGGGCTTCT |
|  | Reverse | GAAGGTGCTGGAGATGG |
| TRINITY_DN115708_c0_g1 | Forward | ATTCAGTATCTCGGAGGGAG |
|  | Reverse | GCTGTTGGGTACATGCC |

**Supplementary Table 2.** Peak time (Pt), name of compounds (NCs), molecular formula (MF), mass-to-charge ratio (m/z), and peak area (PA, %) in N-hexane phase of MPE.

| NCs | Pt (min) | | m/z | MF | PA (%) |
| --- | --- | --- | --- | --- | --- |
| 3-O-talopyranosylmannopyranoside | | 1.5 | 341.1089 | C_12_H_21_O_11_ | 4.69 |
| 3-deoxy-D-manno-octulosonate | | 1.8 | 237.0613 | C_8_H_13_O_8_ | 4.01 |
| 2-acetamido-2-(4-nitrosooxan-4-yl) propanoate | | 2.3 | 243.0983 | C_10_H_15_N_2_O_5_ | 0.83 |
| 5,7-diacetamido-3,5,7,9-tetradeoxy-L-glycero-α-L-manno-non-2-ulopyranosonate | | 2.4 | 333.1305 | C_13_H_21_N_2_O_8_ | 0.36 |
| 4-[3-[2-(4-hydroxyphenyl) ethenyl]-10-methylacridin-10-ium-9-carbonyl] oxy-3,5-dimethylbenzoic acid | | 2.7 | 504.1824 | C_32_H_26_NO_5_ | 0.51 |
| Glutamate-Hydroxyproline dipeptide | | 2.9 | 259.0932 | C_10_H_15_N_2_O_6_ | 4.57 |
| 2-[[4-[naphthalen-2-yl(phenyl)carbamoyl] phenyl] carbamoyl] benzoate | | 3.7 | 485.1514 | C_31_H_21_N_2_O_4_ | 0.96 |
| 4-[bis(1H-indol-3-yl) methyl] benzoate | | 5.7 | 365.1291 | C_24_H_17_N_2_O_2_ | 0.42 |
| 1-[5-(3-propylimidazole-3-gallium-1-yl) heptane-3-yl] pyrrolidine-2-one | | 7.5 | 372.2134 | C_17_H_30_N_3_O | 0.25 |
| 6-(6-Aminohexanamido) hexanoate | | 7.8 | 243.1709 | C_12_H_23_N_2_O_3_ | 0.23 |
| 2-(3,3-dimethyl-2-oxopentanoyl) pyrazolidine-1-carboxylate | | 11 | 241.1190 | C_11_H_17_N_2_O_4_ | 2.19 |
